# Supplementary material for: TMEM106B C-terminal fragments aggregate and drive neurodegenerative proteinopathy
Source: bioRxiv. 2024 Jun 11:2024.06.11.598478. Preprint. [Version 1] doi: 10.1101/2024.06.11.598478 (PMC11195232; doi:10.1101/2024.06.11.598478)
Supplement: Supplement 1 [file NIHPP2024.06.11.598478v1-supplement-1.pdf]

bioRxiv preprint doi: https://doi.org/10.1101/2024.06.11.598478; this version posted June 11, 2024. The copyright holder for this preprint (which was not certified by peer review) is the author/funder. This article is a US Government work. It is not subject to copyright under 17 USC 105 and is also made available for use under a CC0 license.

Supplementary Figures

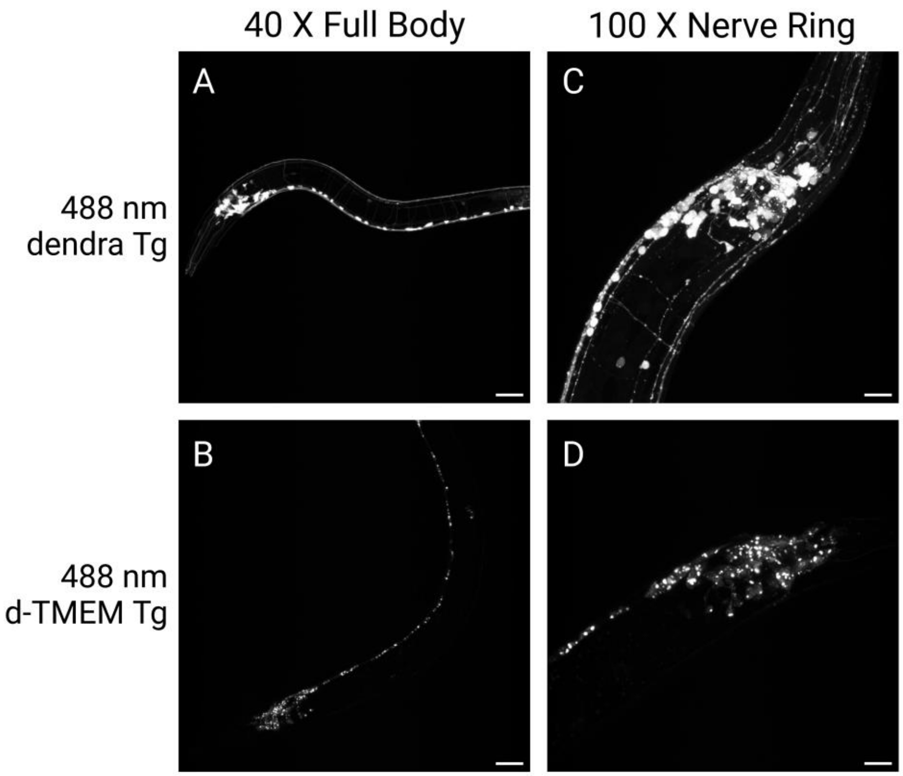

Supplemental Figure S1. Transgenic *C. elegans* model expressing d-TMEM CT at L2 stage of development. 40X and 100X magnification images of L2 staged dendra Tg and d-TMEM CT Tg *C. elegans*. **A-B)** 40X images of full worm body, signal indicating expression of the dendra2 fluorescent protein. Scale bar represents 25 µm. **C-D)** 100X images of nerve ring, signal indicating expression of the dendra2 fluorescent protein. Scale bar represents 10 µm.

bioRxiv preprint doi: https://doi.org/10.1101/2024.06.11.598478; this version posted June 11, 2024. The copyright holder for this preprint (which was not certified by peer review) is the author/funder. This article is a US Government work. It is not subject to copyright under 17 USC 105 and is also made available for use under a CC0 license.

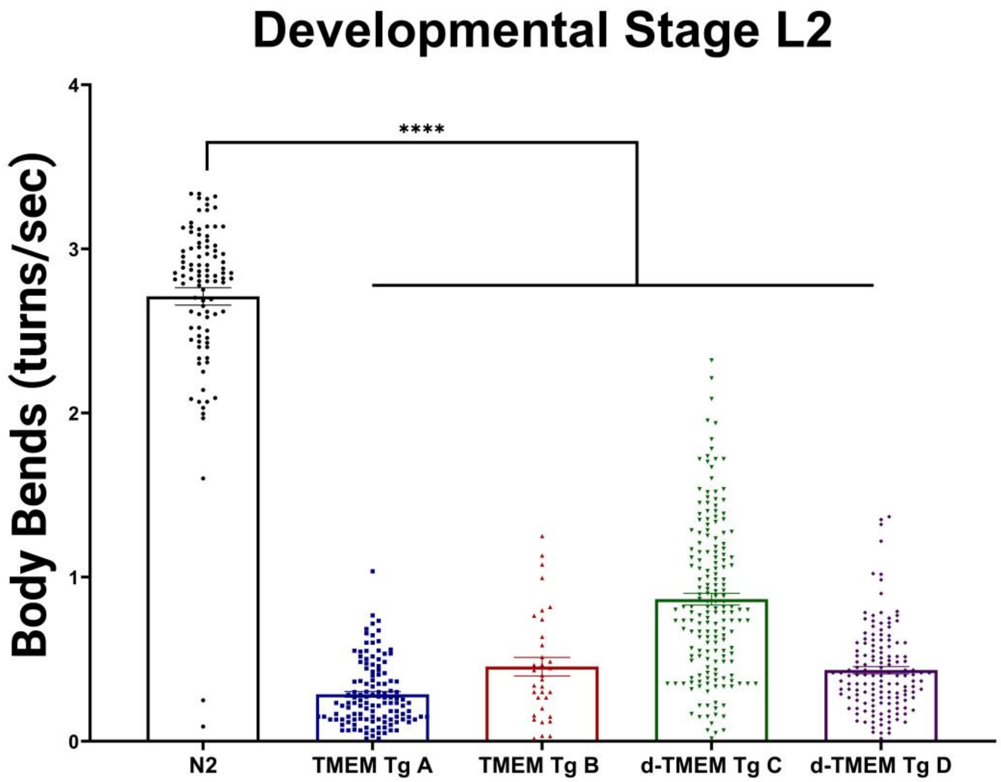

Supplemental Figure S2. TMEM CT expression impairs locomotion at the L2 stage of development. Liquid thrashing assay of TMEM CT and d-TMEM CT strains assessed by computer analysis, mean of replicate averages. n > 34, N = 3 for each strain. *C. elegans* expressing both TMEM CT and d-TMEM CT exhibit significantly impaired thrashing behavior as compared to N2 worms (p<0.0001), indicative of neuronal degeneration. P values denoted as **** for p<0.0001, error bars represent SEM.

bioRxiv preprint doi: https://doi.org/10.1101/2024.06.11.598478; this version posted June 11, 2024. The copyright holder for this preprint (which was not certified by peer review) is the author/funder. This article is a US Government work. It is not subject to copyright under 17 USC 105 and is also made available for use under a CC0 license.

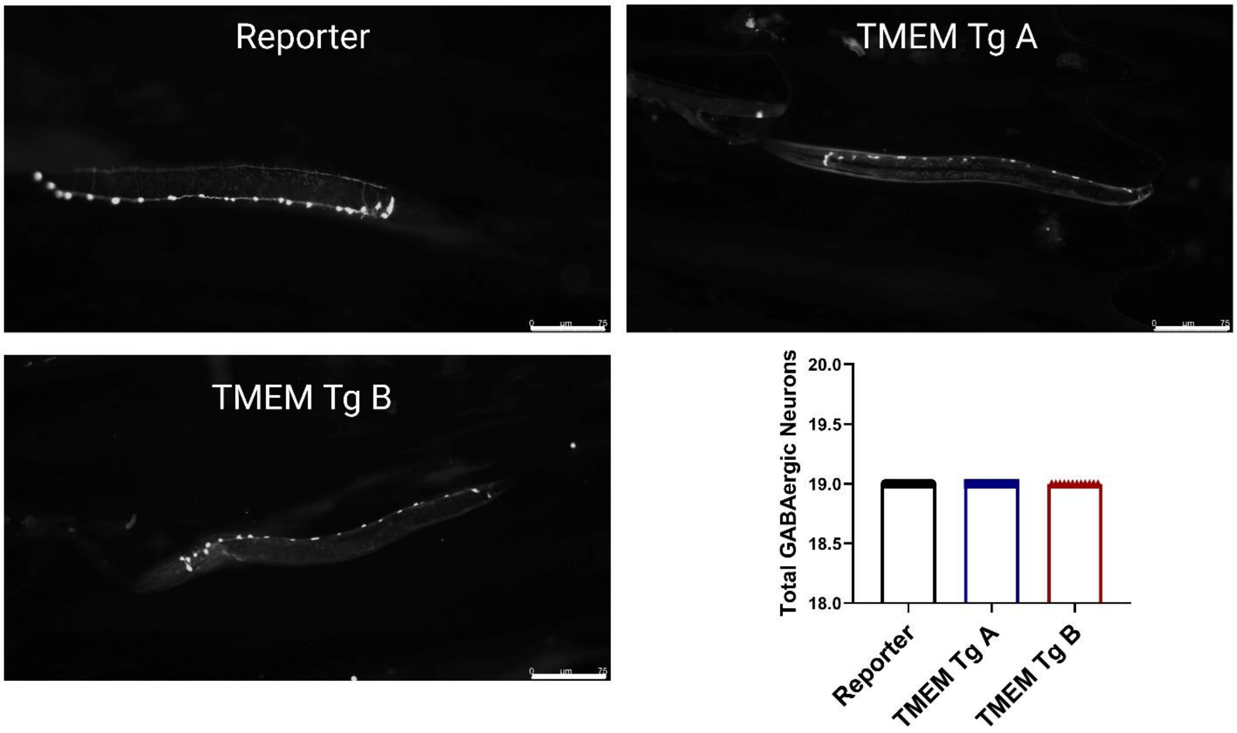

Supplemental Figure S3. TMEM CT Aggregation Does Not Induce Neuronal Loss in L2 Stage *C. elegans*. At the L2 stage of development, neither TMEM Tg A nor TMEM Tg B strain lose any of their 19 GABAergic neurons as visualized by the *unc-47*::GFP reporter (EG1285). Representative images for the reporter strain, TMEM Tg A, and TMEM Tg B and graphical representation of neuronal counts. n > 12 for each strain. Scale bar indicates 75 µM, and error bars represent SEM.

bioRxiv preprint doi: https://doi.org/10.1101/2024.06.11.598478; this version posted June 11, 2024. The copyright holder for this preprint (which was not certified by peer review) is the author/funder. This article is a US Government work. It is not subject to copyright under 17 USC 105 and is also made available for use under a CC0 license.

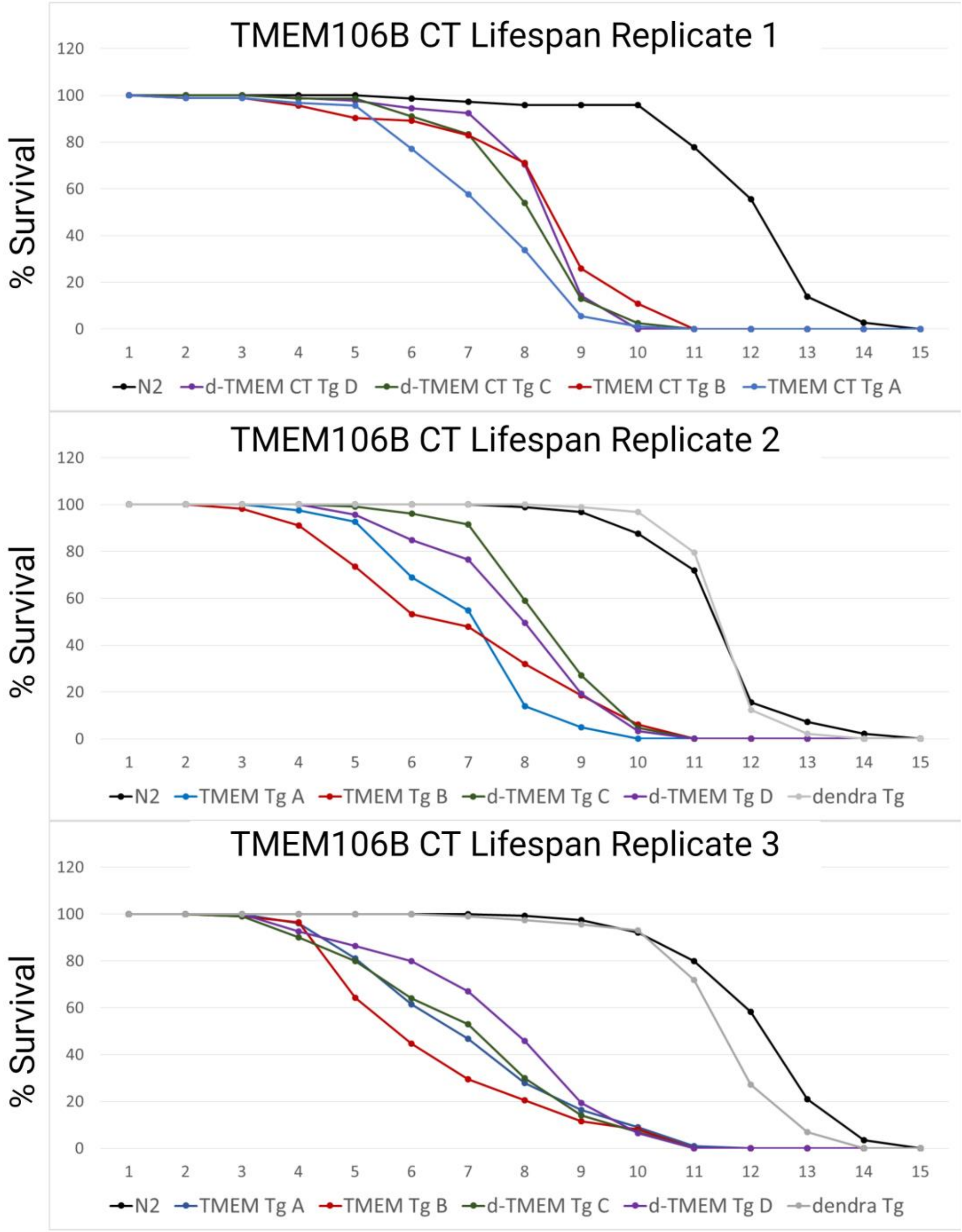

Supplemental Figure S4. TMEM CT Aggregation Severely Decreases Lifespan of *C. elegans.* Lifespan assays of transgenic and wildtype *C. elegans* under FUDR treatment at 25° C. n > 72 worms per strain, N=3. Wild type (N2) worms and transgenic worms expressing only dendra2 had a median survival of around 12 days of adulthood. TMEM CT Tg and d-TMEM CT Tg strains had a significantly reduced median survival ranging from 6 to 8 days of adulthood.

bioRxiv preprint doi: https://doi.org/10.1101/2024.06.11.598478; this version posted June 11, 2024. The copyright holder for this preprint (which was not certified by peer review) is the author/funder. This article is a US Government work. It is not subject to copyright under 17 USC 105 and is also made available for use under a CC0 license.

Rep 1 (1/24-2/8)

Deaths/day	1	2	3	4	5	6	7	8	9	10	11	12	13	14	15
N2	0	0	0	0	0	1	1	1	0	0	13	16	30	8	2
d-TMEM CT Tg D	0	0	0	1	1	3	2	20	51	13	0	0	0	0	0
d-TMEM CT Tg C	0	0	0	1	0	6	6	23	32	8	2	0	0	0	0
TMEM CT Tg B	0	1	0	3	5	1	6	11	42	14	10	0	0	0	0
TMEM CT Tg A	0	1	0	2	1	17	18	22	26	4	1	0	0	0	0

censors/day	1	2	3	4	5	6	7	8	9	10	11	12	13	14	15
N2	13	0	0	4	0	0	0	0	0	0	0	0	0	0	0
d-TMEM CT Tg D	6	0	0	0	0	2	0	0	0	0	0	0	0	0	0
d-TMEM CT Tg C	11	0	0	1	4	0	0	0	0	0	0	0	0	0	0
TMEM CT Tg B	2	0	0	1	3	0	1	0	0	0	0	0	0	0	0
TMEM CT Tg A	5	0	0	2	0	0	0	0	0	0	0	0	0	0	0

Rep 2 (2/8-2/23)

Deaths/day	1	2	3	4	5	6	7	8	9	10	11	12	13	14	15
N2	0	0	0	0	0	0	0	1	2	9	15	54	8	5	2
TMEM Tg A	0	0	0	3	6	29	17	50	11	6	0	0	0	0	0
TMEM Tg B	0	0	2	8	20	23	6	18	15	14	7	0	0	0	0
d-TMEM Tg C	0	0	0	0	1	3	5	35	34	24	5	0	0	0	0
d-TMEM Tg D	0	0	0	0	5	13	10	32	36	19	4	0	0	0	0
dendra Tg	0	0	0	0	0	0	0	0	1	2	17	66	10	2	0

censors/day	1	2	3	4	5	6	7	8	9	10	11	12	13	14	15
N2	0	13	2	0	0	1	5	2	0	0	0	0	0	0	0
TMEM Tg A	0	0	0	0	0	0	0	0	0	0	0	0	0	0	0
TMEM Tg B	1	5	2	0	0	0	0	0	0	0	0	0	0	0	0
d-TMEM Tg C	0	1	1	1	1	0	0	1	0	0	0	0	0	0	0
d-TMEM Tg D	0	1	1	0	0	0	1	0	0	0	0	0	0	0	0
dendra Tg	0	0	0	0	0	0	0	0	0	1	0	0	0	0	0

bioRxiv preprint doi: https://doi.org/10.1101/2024.06.11.598478; this version posted June 11, 2024. The copyright holder for this preprint (which was not certified by peer review) is the author/funder. This article is a US Government work. It is not subject to copyright under 17 USC 105 and is also made available for use under a CC0 license.

Rep 3 (3/14-3/29)

Deaths/day	1	2	3	4	5	6	7	8	9	10	11	12	13	14	15
N2	0	0	0	0	0	0	0	1	2	6	14	25	43	20	4
TMEM Tg A	0	0	0	5	18	24	18	23	14	9	10	1	0	0	0
TMEM Tg B	0	0	1	3	36	22	17	10	10	4	9	0	0	0	0
d-TMEM Tg C	0	0	1	9	10	16	11	23	16	7	7	0	0	0	0
d-TMEM Tg D	0	0	0	8	7	7	14	23	29	14	7	0	0	0	0
dendra Tg	0	0	0	0	0	0	1	2	2	3	24	51	23	8	0

censors/day	1	2	3	4	5	6	7	8	9	10	11	12	13	14	15
N2	0	0	0	2	0	4	0	0	0	0	0	0	0	0	0
TMEM Tg A	3	0	0	0	0	0	0	0	0	0	0	0	0	0	0
TMEM Tg B	8	0	0	1	0	1	0	0	0	0	0	0	0	0	0
d-TMEM Tg C	2	0	7	1	1	0	0	0	0	0	0	0	0	0	0
d-TMEM Tg D	5	0	4	1	0	0	0	0	0	0	0	0	0	0	0
dendra Tg	0	0	2	0	0	0	0	2	0	0	0	1	0	0	0

Supplemental Table S1: Deaths and Censures per Day For *C. elegans* Strains During Lifespan Assay. Worms counted as dead when one did not respond to gentle touch from platinum wire. Worms that burst from FUDR treatment or worms that crawled off of the plate during assay were censored.
